# Comparative genome analysis of marine purple sulfur bacterium *Marichromatium gracile* YL28 reveals the diverse nitrogen cycle mechanisms and habitat-specific traits

**DOI:** 10.1038/s41598-018-36160-2

**Published:** 2018-12-13

**Authors:** Bitong Zhu, Xiaobo Zhang, Chungui Zhao, Shicheng Chen, Suping Yang

**Affiliations:** 1 Department of Bioengineering and Biotechnology, Huaqiao University, Xiamen, 361021 China; 2 Department of Microbiology and Molecular Genetics, Michigan State University, East Lansing, Michigan 48863 USA

## Abstract

Mangrove ecosystems are characteristic of the high salinity, limited nutrients and S-richness. *Marichromatium gracile* YL28 (YL28) isolated from mangrove tolerates the high concentrations of nitrite and sulfur compounds and efficiently eliminates them. However, the molecular mechanisms of nitrite and sulfur compounds utilization and the habitat adaptation remain unclear in YL28. We sequenced YL28 genome and further performed the comparative genome analysis in 36 purple bacteria including purple sulfur bacteria (PSB) and purple non-sulfur bacteria (PNSB). YL28 has 6 nitrogen cycle pathways (up to 40 genes), and possibly removes nitrite by denitrification, complete assimilation nitrate reduction and fermentative nitrate reduction (DNRA). Comparative genome analysis showed that more nitrogen utilization genes were detected in PNSB than those in PSB. The partial denitrification pathway and complete assimilation nitrate reduction were reported in PSB and DNRA was reported in purple bacteria for the first time. The three sulfur metabolism genes such as oxidation of sulfide, reversed dissimilatory sulfite reduction and *sox* system allowed to eliminate toxic sulfur compounds in the mangrove ecosystem. Several unique stress response genes facilitate to the tolerance of the high salinity environment. The CRISPR systems and the transposon components in genomic islands (GIs) likely contribute to the genome plasticity in purple bacteria.

## Introduction

Purple bacteria are anoxygenic and phototrophic bacteria, including purple sulfur bacteria (PSB) and purple non-sulfur bacteria (PNSB). They are ubiquitously distributed in different natural environments with versatile metabolism potentials. Purple bacteria also serve as models for clarifying the biogeochemistry of C, N, S and Fe in the earth evolution^1^. YL28, a member of PSB, was isolated from mangrove special ecosystem^2^. It synthesized abundant rhodopin (not spirilloxanthin) carotenoid component under anaerobic in the light condition. It was also capable of using reduced sulfur compounds, nitrogen compounds or molecular hydrogen as electron donors^2^. Moreover, YL28 utilized ammonium, nitrite or nitrate as the sole nitrogen source for phototrophic growth. It is interesting that it was found to completely remove nitrite (up to 200 mg/L)^3,4^ and perform simultaneous heterotrophic nitrification and denitrification under anaerobic conditions^5^.

Nitrogen is one of the most abundant elements on earth. It comprises the majority of earth’s atmosphere and functions as one of the primary nutrients^6^. Nitrogen cycle is the most complex biogeochemical one in the biosphere^7,8^. However, the nitrogen cycle has been drastically disrupted because of overexploitation and modern agricultural activities^9^. Nearly half of the nitrogen reaches the coastal ocean via river input and/or atmospheric deposition^10^. This leads to extensive eutrophication of waters and coastal zones and increases inventories of the potent greenhouse gas (such as nitrous oxide). The elevated concentrations of NH_3_-N and NO_2_-N (the major pollutants) are problematic in aquatic ecosystems^11^.

Up to 10 nitrogen cycle pathways were reported^9^. For examples, purple bacteria have three nitrogen reduction cycles (reduced nitrogen compounds as electron acceptor, such as nitrogen fixation, nitrate assimilation, denitrification) and one nitrogen oxidation cycle (nitrite oxidation to nitrate as electron donor for photosynthesis)^12–15^. However, nitrification or dissimilatory nitrate reduction to ammonium (DNRA) pathway has not been reported in purple bacteria yet. Denitrification and assimilation nitrate reduction cycles were only reported in PNSB, but unknown in PSB^16^. Currently, the molecular mechanism of nitrogen cycles in purple bacteria remain unclear^17–19^. To elucidate nitrogen cycles in purple bacteria, we sequenced the genome of YL28 as a representative strain, compared the nitrogen cycle genes to other 35 sequenced purple bacteria. The genes involved in the sulfur metabolism, salt tolerance and the stress response were also investigated in details to gain the insights of the surviving mechanisms of YL28 in the highly selective environments.

## Results

### Genome features and phylogenic inference

The general features of genome of YL28 and the other 35 sequenced purple bacteria were presented in Table 1. Removal of short contigs and sequences with potential contamination resulted in 120 contigs with a N_50_ of 93,809 bp. The largest contig size was 176,489 bp. The assembled genome comprised 3.8 million nucleotides with a GC content of 68.84%. Gene annotation was carried out by the NCBI Prokaryotic Genome Annotation Pipeline (PGAP). A total of 3,361 genes yielded a coding capacity of 3.3 million nucleotides (genes/genome, 86.4%). There were at least 69 RNA sequences, including 56 tRNAs, 9 rRNAs, and 4 function unknown RNAs in the genome. Among the protein-encoding genes, 1,752 of them could be assigned putative functions while 1,612 were predicted to encode hypothetical proteins. At least 1,671 proteins were assigned to 25 different functional categories with 368 subsystems using SEED subsystems by RAST analysis (Supplementary Fig. SF1). 40 genes involved in nitrogen metabolism were found (Supplementary Fig. SF1). Compared to its relative *M*. *purpuratum* 984, YL28 has more genes for nitrogen metabolism, photosynthesis and sulfur metabolism (Supplementary Fig. SF2, Tables 2 and 3).

**Table 1:** The genome characteristics of purple bacteria used in this study.

Organisms	Chromosome	Plasmid	Size (Mb)	Chr 1 (Mb)	Chr 2 (Mb)	G + C %	Protein	gene	Accession
YL28	1	—	3.84	3.84	—	68.9	3,188	3,360	LSYU00000000.1
*Thiocystis violascens* DSM 198	1	—	5.02	5.02	—	62.6	4,261	4,465	CP003154.1
*Allochromatium vinosum* DSM 180	1	2	3.67	3.53	—	64.4	3,148	3,262	CP001896.1
*Marichromatium purpuratum* 984	1	—	3.78	3.78	—	67.9	3,159	3,272	EU850807.1
*Ectothiorhodospira sp*. BSL-9	1	—	3.55	3.55	—	63	3118	3,249	CP011994.1
*Thioflavicoccus mobilis* 8321	1	1	4.13	4.05	—	65.5	3462	3,602	NR102479.1
*Halorhodospira halophila* SL1 *extremly halophilic*	1	—	2.68	2.68	—	68	2375	2,458	CP000544.1
*Rhodopseudomonas palustris* CGA009	1	1	5.47	5.46	—	65	4,820	4,898	BX572608.1
*Rhodopseudomonas palustris* HaA2	1	—	5.33	5.33	—	66	4,683	4,772	CP000250.1
*Rhodopseudomonas palustris* BisB18	1	—	5.51	5.51	—	65	4,886	5,016	CP000301.1
*Rhodopseudomonas palustris* BisB5	1	—	4.89	4.89	—	64.8	4,397	4,492	CP000283.1
*Rhodopseudomonas palustris* BisA53	1	—	5.51	5.51	—	64.4	4,878	4,972	CP000463.1
*Rhodopseudomonas palustris* TIE-1	1	—	5.74	5.74	—	64.9	5,246	5,382	CP001096.1
*Rhodopseudomonas palustris* DX-1	1	—	5.4	5.4	—	65.4	4,917	5,082	EU221583.1
*Rhodoplanes* sp. Z2-YC6860	1	—	8.19	8.19	—	63.5	7443	7,606	GQ369128.3
*Blastochloris viridis*	1	—	3.73	3.72	—	67.9	3107	3,230	D14430.2
*Blastochloris viridis* ATCC19567	1	—	3.73	3.72	—	67.9	3104	3,238	D2 5314.1
*Blastochloris viridis* DSM_133	1	—	3.72	3.72	—	67.9	3114	3,236	HQ009851.1
*Rhodospirillum rubrum* ATCC 11170	1	1	4.41	4.35	—	65.3	3,838	3,917	CP000230.1
*Rhodospirillum rubrum* F11	1	—	4.35	4.35	—	65.4	3,878	3,946	CP003046.1
*Rhodospirillum centenum* SW (Rhodocista centenaria)	1	—	4.36	4.36	—	70.5	4,003	4,102	CP000613.2
*Pararhodospirillum photometricum* DSM 122	1	—	3.88	3.88	—	64.7	3,117	3,434	HE663493.1
*Rhodomicrobium vannielii* ATCC 17100	1	—	4.01	4.01	—	62.2	3,565	3,793	CP002292.1
*Rhodobacter sphaeroides* 2.4.1	2	5	4.6	3.19	0.94	68.8	4,242	4,370	CP000144.2
*Rhodobacter sphaeroides* MBTLJ-8	2	4	4.66	3.19	0.94	68.7	4204	4,362	CP012960.1
*Rhodobacter sphaeroides* MBTLJ-13	2	5	4.65	3.19	0.94	68.8	4200	4,358	CP015210.1
*Rhodobactersphaeroides* ATCC 17029	2	1	4.49	3.15	1.22	69	4,131	4,254	CP000577.1
*Rhodobacter sphaeroides* ATCC 17025	1	5	4.56	3.22	—	68.2	4,333	4,466	CP000661.1
*Rhodobacter sphaeroides* KD131	2	2	4.71	3.15	1.3	69.1	4,569	4,638	CP001151.1
*Rhodoferax ferrireducens T118*	1	1	4.97	4.71	—	59.6	4,436	4,554	CP000267.1
*Rhodobacter capsulatus* SB 1003	1	1	3.87	3.74	—	66.6	3,642	3,740	CP001312.1
*Rhodobacter* sp. LPB0142	1	3	3.78	3.46	—	66	3530	3,660	KX066852.2
*Rhodovulum sulfidophilum* DSM1374	1	2	4.34	4.13	—	66.9	3876	4,002	CP015418.1
*Rhodovulum sulfidophilum* DSM2351	1	3	4.73	4.45	—	66.9	4155	4,340	D16430.1
*Rhodovulum sulfidophilum* SNK001	1	1	4.19	4.08	—	66.8	3746	3,873	CP015421.1
*Rubrivivax gelatinosus* IL144	1	—	5.04	5.02	—	71.2	4505	4,591	AB016167.1

**Table 2:** The key enzymes responsible for different nitrogen metabolism in purple bacteria.

Species	Fermentative nitrate reduction		Aissimilatory nitrate reduction		Denitrification				Nitrogen fixation
	*nar*GHIJ/napAB	*nir*BD*/nrf*AH	*nar*B/NR*/nas*AB	NIT-6*/nir*A	*nar*GHIJ*/nap*AB	*nir*K*/nir*S	*nor*BC	*nos*Z	*nif*DKH*/anf*G
*R*. *palustris* CGA009				*nir*A		*nir*K	*nor*BC	*nos*Z	*nif*DKH*/anf*G
*R*. *palustris* HaA2		*nir*BD	*nas*AB	*nir*A					*nif*DKH
*R*. *palustris* BisB18	*nap*AB				*nap*AB			*nos*Z	*nif*DKH*/anf*G
*R*. *palustris* BisB5									*nif*DKH*/anf*G
*R*. *palustris* BisA53		*nir*BD	*nas*AB	*nir*A		*nir*K	*nor*BC	*nos*Z	
*R*. *palustris* TIE-1				*nir*A		*nir*K	*nor*BC	*nos*Z	
*R*. *palustris* DX-1				*nir*A		*nir*K	*nor*BC	*nos*Z	*nif*DKH*/anf*G
*Rhodoplanes sp*. Z2-YC6860	*nar*GHIJ	*nir*BD	*nas*AB						
*B*. *viridis*									*nif*DKH*/anf*G
*B*. *viridis* ATCC19567									*nif*DKH*/anf*G
*B*. *viridis* DSM_133									*nif*DKH*/anf*G
*R*. *vannielii* ATCC 17100	*nap*AB				*nap*AB		*nor*BC		*nif*DKH
*R*. *sphaeroides* 2.4.1	*nap*AB				*nap*AB	*nir*K	*nor*BC		*nif*DKH
*R*. *sphaeroides* ATCC17029							*nor*BC		*nif*DKH
*R*. *sphaeroides* ATCC17025					*nap*AB	*nir*K	*nor*BC	*nos*Z	*nif*DKH
*R*. *sphaeroides* KD131						*nir*K	*nor*BC	*nos*Z	
*R*. *capsulatus* SB1003								*nos*Z	*nif*DKH*/anf*G
*R*. *sphaeroides* MBTLJ-13	*nap*AB				*nap*AB		*nor*BC		*nif*DKH
*R*. *sphaeroides* MBTLJ-8	*nap*AB				*nap*AB		*nor*BC		*nif*DKH
*R*. *rubrum* ATCC11170									*nif*DKH*/anf*G
*R*. *rubrum* F11									*nif*DKH*/anf*G
*R*. *centenum* SW	*nap*AB				*nap*AB		*nor*BC	*nos*Z	*ni*fDKH
*P*. *photometricum* DSM 122									*nif*DKH*/anf*G
*R*. *vannielii* ATCC17100							*nor*BC		*nif*DKH*/anf*G
*R*. *ferrireducens* T118	*nar*GHIJ	*nir*BD	*nas*AB		*nar*GHIJ		*nor*BC	*nos*Z	
*R*. *sulfidophilum* DSM1374									*nif*DKH
*R*. *sulfidophilum* DSM2351									*nif*DKH
*R*. *sulfidophilum* SNK001									*nif*DKH
*R*. *gelatinosus* IL144						*nir*K	*nor*BC	*nos*Z	*nif*DKH
*T*. *violascens* DSM198	*nap*AB				*nap*AB				*nif*DKH
*A*. *vinosum* DSM180				*nirA*			*nor*BC		*nif*DKH
*M*. *purpuratum* 984		*otr*	*nas*AB			*nir*K	*nor*BC		*nif*DKH
YL28		*nrf*AH	*nas*AB	*nirA*		*nir*S	*nor*BC		*nif*DKH
*H*. *halophila* SL1	*nar*GHIJ	*nrf*AH			*nar*GHIJ		*nor*BC		*nif*DKH
*Ectothiorhodospira sp*. BSL-9		*nrf*AH							*nif*DKH
*T*. *mobilis* 8321									*nif*DKH

**Table 3:** The key enzymes responsible for different sulfur metabolism in purple sulfur bacteria (PSB).

Species	Oxidation of sulfide	Reversed dissimilatory sulfite reductase			Sox system			
	FCC/SQR	DsrAB	AprAB	Sat	SoxB	SoxY/SoxZ	SoxA/SoxX	SoxC/SoxD
*A*. *vinosum* DSM180	SQR	DsrAB	AprAB	Sat	SoxB	SoxY/SoxZ	SoxA/SoxX	—
*Ectothiorhodospira sp*. BSL-9	FCC	—	—	Sat	—	—	—	—
*H*. *halophila* SL1	SQR	DsrAB	—	—	SoxB	SoxY/SoxZ	SoxA	—
**YL28**	SQR	DsrAB	AprAB	Sat	SoxB	SoxY/SoxZ	SoxA/SoxX	SoxD
*M*. *purpuratum* 984	SQR	DsrAB	—	—	SoxB	SoxY/SoxZ	SoxA/SoxX	SoxD
*T*. *violascens* DSM198	FCC	DsrAB	AprAB	Sat	SoxB	SoxY/SoxZ	SoxA/SoxX	—
*T*. *mobilis* 8321	FCC	DsrAB	AprAB	Sat	—	—	—	—

No plasmid sequences were discovered in YL28. However, at least one was found in *Rhodovulum* spp, *Rhodobacter* spp, *Allochromatium* spp, *R*. *rubrum* ATCC 11170, *T*. *mobilis* 8321 and *R*. *palustris* CGA009 (Table 1). Moreover, most of the *Rhodobacter* strains possesses up to two chromosomes. The genomic size of the purple bacteria ranges from 2.6 to 5.4 Mb. Except for *T*. *mobilis* 8321 and *T*. *violascens* DSM 198, the genome size in PSB is smaller than that in PNSB (Table 1). Overall, genome size in most of PSB is smaller than 4.0 MB, with the smallest one (2.68 Mb) in *Halorhodospira halophila* SL1. Except for *Blastochloris*, genome size of PNSB is generally larger than 4.0 MB; among them, *Rhodoplanes* sp. Z2-YC6860 has the largest genome size (8.19 Mb). Collectively, a high GC content (>55%) is often observed in purple bacteria.

### Phylogenic inference

**Figure 1:**
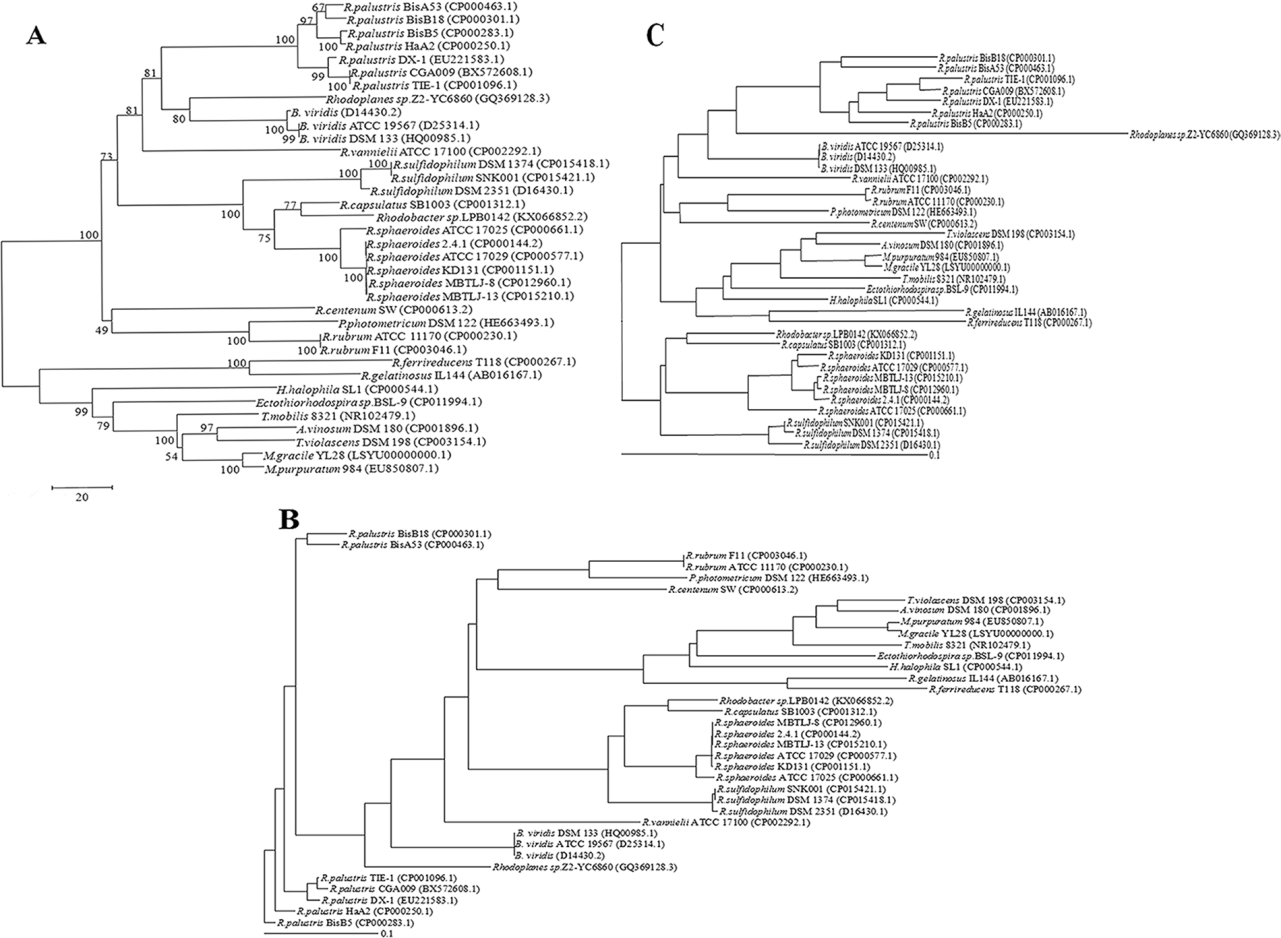
Phylogenetic analyses between PSB and PNSB. The NJ algorithm tree of 16S rRNA genes of 36 strains from purple bacteria by the MEGA 6.06 (**A**); the Mauve guide tree of the 35 strains based on whole-genomic similarity at the nucleotide level through multiple genome comparison tool Mauve (**B**); the NJ tree of 77 conserved proteins shared among the 36 strains by the BPGA (**C**).

The phylogenetic analysis based on 16S rDNA sequence (Fig. 1A) showed that PSB and PNSB derived from a common ancestor and formed two clades, which was consistent with the previous studies^20^. 28 PNSB strains were clustered into three subdivisions: the first one consisted of *Rhodopseudomonas*, *Blastochloris*, *Rhodoplanes* and *Rhodomicrobium vannielii* ATCC 17100; the second one included *Rhodobacter* (freshwater species) and *Rhodovulum* (marine species); the third one had *Rhodospirillum* and *Rhodocista*. 7 PSB species were clustered together on the same clade. Both the phylogenetic trees based on whole genome or core genome (Fig. 1B,C) showed that some PSB species were included in PNSB. Interestingly, *Rubrivivax gelatinosus* IL144 (a member of PNSB) was the closest phylogenetic neighbors of PSB based on 16S rDNA sequence and core genome.

### Gene repertoire of PSB

**Figure 2:**
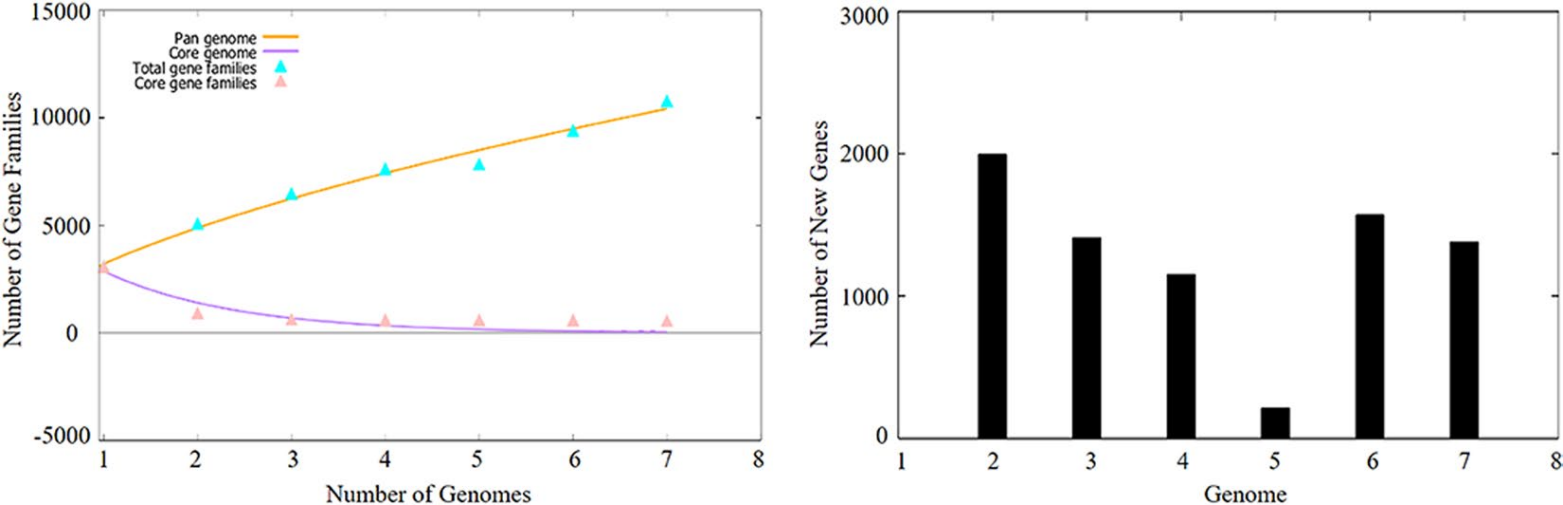
Pan, core and singleton genome evolution according to the number of selected purple bacteria genomes. (**A**) Number of genes (core genome and Pan-genome) for the selected purple bacteria genomes sequentially added. (**B**) Number of unique genes for the selected purple bacteria genomes sequentially added.

The power trend line has not reached the plateau (Fig. 2A), demonstrating that PSB displays an open accessory-genome. The core genome analysis of PSB shows that the numbers of shared genes decreases with the addition of the input genomes and is predicted to converge against 550 (Fig. 2A). Singleton development plot data indicates that up to 1594 new genes are expected with each newly added genome (Fig. 2B). The core genomes for the seven PSB species contain at least 539 CDSs per genome. The current core genome represents the PSB quite well. YL28 and *M*. *purpuratum* 984 share at least 2869 genes (data not shown); only 241 and 210 genes uniquely occur in YL28 and 984, respectively. The shared gene numbers in genome between YL28 and other six PSB species range from 732 to 2869 genes (data not shown).

### Nitrogen cycle genes in YL28 and purple bacteria

**Figure 3:**
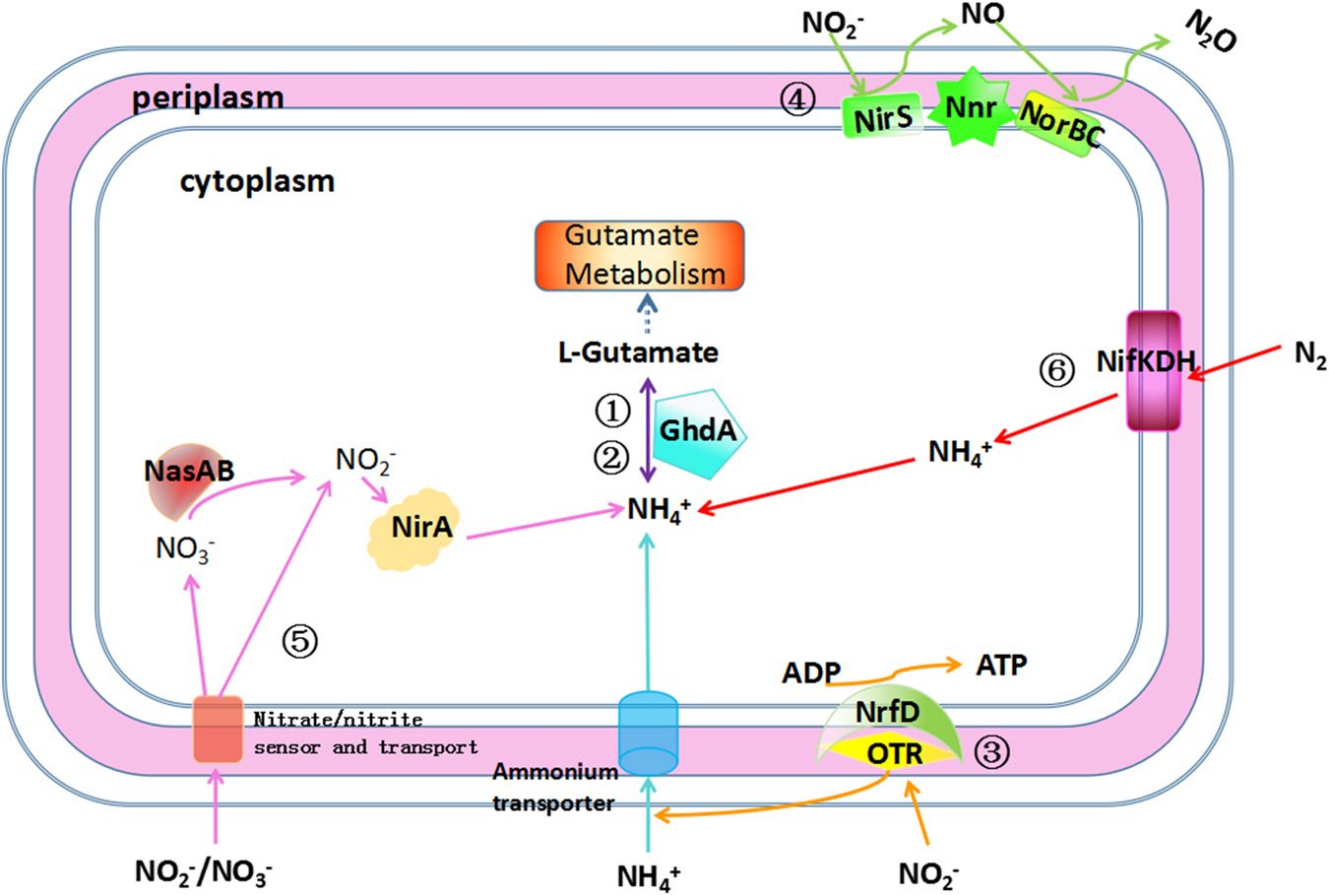
The model of nitrogen metabolism in *M*. *gracile* YL28. There are six nitrogen metabolic pathways in YL28 whose key enzyme confirmed by CDART and CDD. (**A**) Ammonification pathway; (**B**) ammonium assimilation pathway; (**C**) fermentative nitrate reduction; (**D**) denitrification; (**E**) assimilation nitrate reduction; (**F**) nitrogen fixation.

YL28 possesses six nitrogen cycle pathways (Fig. 3 and Table 2). Two genes encoding NirS and NorBC (the key enzymes for nitrite reduction) exists in YL28. These two enzymes in YL28 possibly contribute to transform toxic nitrite into lower cytotoxic N_2_O by denitification^5^. The genes encoding NasAB and NirA (the key enzymes for assimilation nitrite reduction) were also detected in YL28, suggesting that it had the complete nitrate reduction ability. YL28 has the genes encoding Nrf which is critical in fermentative nitrate reduction (DNRA). However, the DNRA pathway in YL28 may be incomplete due to the lack of entire complement enzymes. Ammonium assimilation and ammonification related genes were detected in all purple bacteria. Additional four nitrogen cycle pathways display diverse metabolism traits in examined strains (Table 2). The presence of genes encoding NifDKH key enzymes in all purple bacteria shows this group of bacteria has nitrogen fixation potentials. Interestingly, the alternative nitrogenase (AnfG) from *Methanosarcina* was only found in PNSB but not in PSB^21^.

### Sulfur metabolism genes in YL28

YL28 has genes invovled in at least three sulfur metabolism pathways such as oxidation of sulfide, reversed dissimilatory sulfite reduction and sox system (Table 3). The *sqr* and the *dsr* gene cluters (*dsrABEFHCMKLJOPNR*) are the key enzyme genes for the oxidation of sulfide and the reversed dissimilatory sulfite reduction. These process oxidize toxic sulfide into S^0^ or sulfate^20^. In addition, *sox* gene clusters in YL28 shows that thiosulfate is possbly converted into S^0^ or sulfate by the truncated Sox system. Moreover, the presence of *aprAB* and *sat* genes (encoding adenylylsulfate reductase, sulfate adenylyltransferase, respectively) suggests that YL28 possibly possesses an alternative sulfite oxiditon pathway (converting toxic sulfite into sulfate).

### Halo-tolerance

YL28 possessed a gene cluster involved in the salt-alkali tolerance (*nhaABCDEFG*, encoding Na^+^/H^+^ antiporter). Moreover, a novel putative Na^+^/H^+^ antiporter gene (*duf* 2062) exsit in YL28. The unique stress response subsystem in YL28 was listed in Table 4. There are six unique proteins participating in choline and betaine uptake and betaine biosynthesis (betaine aldehyde dehydrogenase, choline dehydrogenase), synthesis of osmoregulated periplasmic glucans (phosphoglycerol transferase I, cation channel protein), heavy metal resistence (DNA-binding heavy metal response regulator, heavy metal sensor histidine kinase).

**Table 4:** The unique stress response protein in YL28.

Category	Subcategory	Subsystem	Role
Virulence, Disease and Defense	Resistance to antibiotics and toxic compounds	Cobalt-zinc-cadmium resistance	DNA-binding heavy metal response regulator
	Resistance to antibiotics and toxic compounds	Cobalt-zinc-cadmium resistance	Heavy metal sensor histidine kinase
	Resistance to antibiotics and toxic compounds	Cobalt-zinc-cadmium resistance	cation channel protein
Stress Response	Osmotic stress	Choline and Betaine Uptake and Betaine Biosynthesis	Betaine aldehyde dehydrogenase (EC 1.2.1.8)
	Osmotic stress	Choline and Betaine Uptake and Betaine Biosynthesis	Choline dehydrogenase (EC 1.1.99.1)
	Osmotic stress	Synthesis of osmoregulated periplasmic glucans	Phosphoglycerol transferase I (EC 2.7.8.20)

### Genome islands (GIs) of YL28

**Figure 4:**
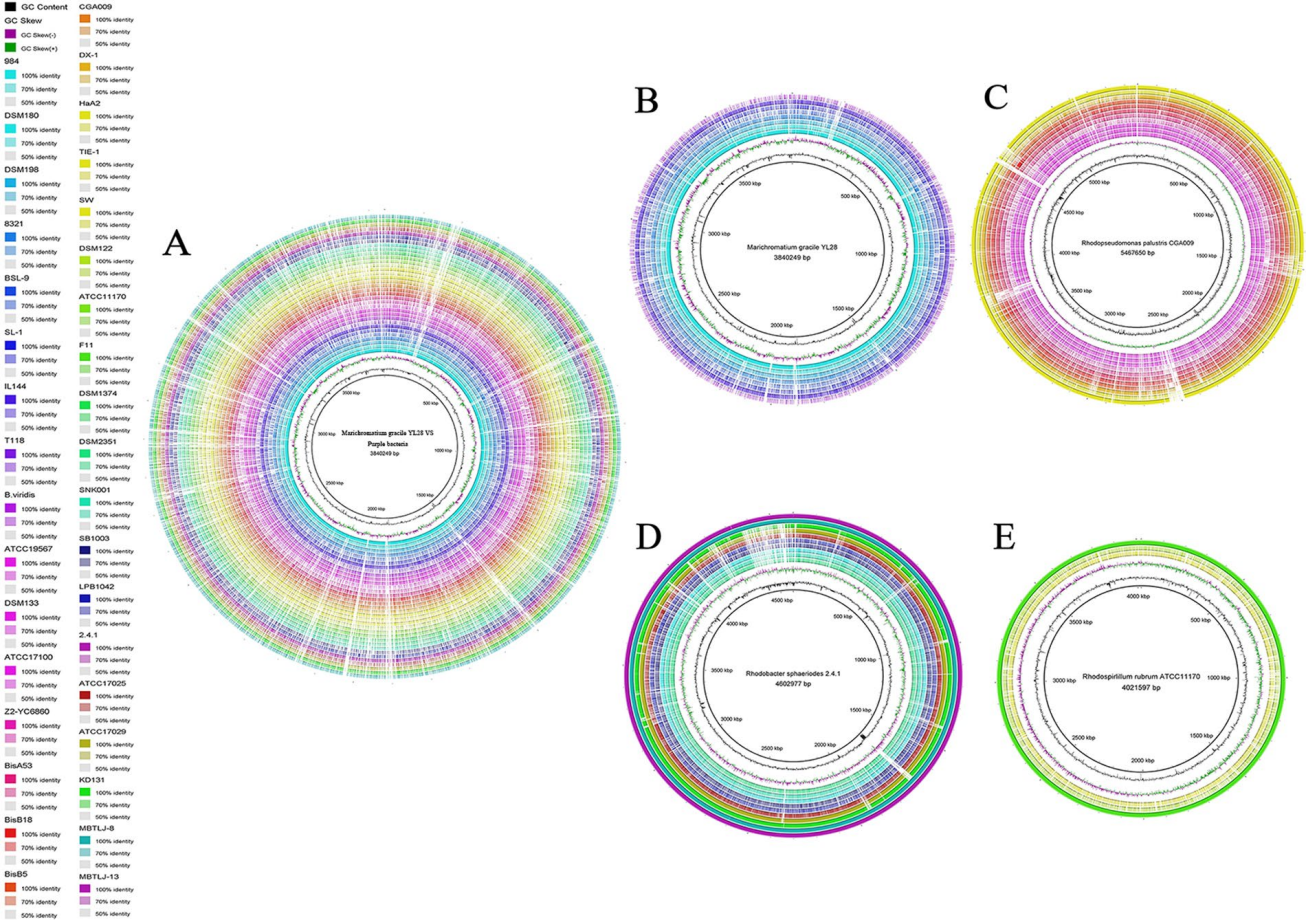
Whole-genome comparisons in three genera of purple bacteria. The color intensity in each ring represents the BLAST match identity. (**A**) Whole-genome comparison of all the strains considered in this work; (**B**) Whole-genome comparisons of strains of purple sulfur bacteria and T118, IL144. The color intensity in each ring represents the BLAST match identity. from inner to outer ring: *M*. *purpuratum* 984, *A*. *vinosum* DSM 180, *T*. *violascens* DSM 198, *T*. *mobilis* 8321, *Ectothiorhodospira* sp. BSL-9, *H*. *halophila* SL1, *R*. *gelatinosus* IL144, *R*. *ferrireducens* T118. (**C**) Whole-genome comparisons of 12 strains of purple non-sulfur bacteria. The color intensity in each ring represents the BLAST match identity. from inner to outer ring: *R*. *palustris* BisA53, BisB18, BisB5, DX-1, HaA2, TIE-1, *B*. *viridis*, *B*. *viridis* ATCC19567, *B*. *viridis* DSM133, *Rhodoplanes* sp. Z2-YC6860, *R*. *vannielii* ATCC 17100. (**D**) Whole-genome comparisons 11 strains of two gena in *Rhodobacter* and *Rhodovulum* in purple non-sulfur bacteria. The color intensity in each ring represents the BLAST match identity from inner to outer ring: *R*. *capsulatus* SB 1003, *Rhodobacter* sp. LPB0142, *R*. *sphaeroides* ATCC 17025, *R*. *sphaeroides* ATCC 17029, *R*. *sphaeroides* KD131, *R*. *sphaeroides* MBTLJ-13, *R*. *sphaeroides* MBTLJ-8, *R*. *sulfidophilum* DSM1374, *R*. *sulfidophilum* DSM2351, *R*. *sulfidophilum* SNK001; (**E**) whole-genome comparisons in *Rhodobacter*, from inner to outer ring: *R*. *rubrum* F11, *R*. *centenum* SW, *P*. *photometricum* DSM 122.

At least 9 GIs were identified with YL28 (Fig. 4A,B and Supplemental Table ST1) by both IslandPick and IslandPath-DIMOB methods. The GI size ranges from 8 to 31.3 Kb (Supplemental Table ST1). These GIs were noted as follows: the mobile element proteins, transposase, phage structure proteins, integration host factor, proteins involved in CRISPR system, 5-methylcytosine-specific restriction related enzyme, cephalosporin hydroxylase, flagellum synthesis component (CheABR), carbohydrate/nitrogen metabolism proteins (phenylalanyl-tRNA metabolism, Threonyl-tRNA biosynthesis, amylomaltase, asparagine synthetase, glycosyl transferase, aspartate aminotransferase), DNA replication and proofreading systems (DNA recombination, repair DNA recombination and repair protein RecF Type II restriction enzyme, ATP-dependent endonuclease of the OLD family, ATP-dependent DNA helicase pcrA, DNA processing chain A) (Fig. 4A,B and Supplemental Table ST1).

Among these predicted GIs, GI-IX has the largest size (31.3 kb) in YL28. Notably, GI-IX has many DNA metabolic related genes including DNA recombination and repair protein RecF, ATP-dependent endonuclease of the OLD family, ATP-dependent DNA helicase *pcr*A, ATP-dependent DNA helicase RecQ, DNA processing chain A and type II restriction enzyme. It also contains some genes encoding diverse enzymes such as aspartate aminotransferase, methylase and ATP-dependent protease. The second largest GI (GI-II) carries a chemoreceptor gene cluster including the *cheA*, *cheB*, *cheR* and a gene encoding the methyl-accepting chemotaxis protein. Moreover, GI-II has the predicted two-component hybrid sensor and regulator, 4-alpha-glucanotransferase (amylomaltase), and mobile element protein (Supplemental Table ST1). Immediately downstream of GI-II, there are genes involved in the DNA metabolism such as 5-methylcytosine-specific restriction related enzyme, DNA helicase, phosphatase 2C homolog and 5-methylcytosine-specific restriction related enzyme. The size of other GIs is smaller than GI-IX.

There are five GIs in *Rhodopseudomonas*, *Blastochloris* and *Rhodoplanes* (Supplemental Table ST2). These GIs carry nitrogen-fixing genes (nitrogenase gene and alternative nitrogenase gene), LysR family of proteins transcriptional regulation, glycosyltransferase protein family and arsenic resistance (ArsH). There is an alternative nitrogenase (AnfG) in a GI region of *R*. *palustris*, which replaces nitrogenase (NifDKH) for nitrogen fixation^22^. At least 10 GIs are predicted in *Rhodobacter* (Supplemental Table ST3). Among these GIs, there are many genes involved in nitrogen metabolism including a nitrogen-fixing island (nitrogenase genes, oxidoreductase/nitrogenase component), a nitrogen-rich^23^ island (nitrate/sulfonate/bicarbonate ABC transporter, nitrogen regulatory protein P-II), and a solid-island^24^ (metallophosphoesterase). Moreover, some GIs were found to have several genes that involved in sulfur metabolism, biosynthesis of flagella, phage infection area and BadM/Rrf2 family of transcriptional regulatory protein. *Rhodobacter* possibly acquires nitrogen-fixation and the sulfur metabolism by the horizontal gene transfer (HGT). At least 11 GIs were predicted in genus *Rhodospirillum* (Supplemental Table ST4). Among those GIs, *Rhodospirillum* has gene elements including CRISPR, sulfide metabolism, arsenic resistance and the DNA and ribosomal protein synthesis, allowing this bacterium to survive in toxic environments under certain concentration of arsenic and sulfide.

### Synteny analysis

A good collinearity relationship was shown in PSB using the synteny plots analysis (Supplementary Fig. SF3, YL28 as the reference genome). YL28, *T*. *violascens* DSM 198, *A*. *vinosum* DSM 180, *T*. *mobilis* 8312 and *M*. *purpuratum* 984 have the closer relationship than do *Ectothiorhodospira* sp. BSL-9 and *H*. *halophila* SL1. When *R*. *palustris* CGA009, *R*. *sphaeroides* 2.4.1 or *R*. *rubrum* ATCC 11170 was selected as a reference sequence, PNSB members showed a diverse collinearity relationship (Supplementary Figs SF4–SF6). Three groups are divided: the first one consists of the 4 genera including *Rhodopseudomonas*, *Blastochloris*, *Rhodoplanes*, and *Rhodomicrobium* (Fig. SF4); the second one consists of the 2 genera *Rhodobacter* and *Rhodovillum* (Fig. SF5); the third one only contains *Rhodospirillum* (except *R*. *centenum* SW) (Fig. SF6). Interestingly, a good collinearity was found between *R*. *ferrireducens* T118 (a non-phototrophic member) and PSB. Instead, a relatively poor collinearity was presented among PSB, *R*. *gelatinosus* IL144 and *R*. *centenum* SW. Collectively, the synteny pattern is consistent with the whole genome phylogenetic tree (Fig. 1B). The poorest collinearity relationship was shown between YL28 and *R*. *gelatinosus* IL144.

## Discussion

The interfaces of mangrove locate between land and sea in the tropical and the sub-tropical latitudes^25^. PSB and PNSB are predominantly detected in the mangrove ecosystems. The two bacterial groups significantly contribute to the primary productivity of coastal seaboards and the food-web dynamics of various tropical coastline ecosystems^26,27^. The high salinity, limited nutrients and S-richness sulfate concentrations often occur in the niche^25^. However, it remains unclear about how the mangrove-associated microorganisms survive in the mangrove ecosystems and contribute to the host physiology. Particularly, the nitrogen utilization mechanisms in PSB and PNSB have not well studied. In this study, we sequenced the genome of YL28, investigated the nitrogen cycle pathways and compared them to other purple bacteria. Our study will contribute to elucidate bacterial surviving mechanisms in the special mangrove ecosystem.

YL28 utilized ammonium, nitrite or nitrate as the sole nitrogen source for phototrophic growth^3–5^. In the present study, our results reveal that the strong ability for nitrate and nitrite utilization may be due to the six nitrogen metabolic pathways. Ammonium is preferred nitrogen sources by all species of purple bacteria^28^. YL28 may grow on ammonium by the ammonium assimilation pathway, and converted organic nitrogen into ammonium by ammonification. Moreover, YL28 utilized N_2_ as nitrogen source to support cell growth by nitrogen fixation when the absence of ammonium happened. It is interesting that YL28 grew chemoheterotrophically using the nitrate and/or nitrite as electron acceptor. The denitrification and/or fermentative nitrate reduction and/or assimilation nitrate reduction pathways may contribute to this process under the anoxic condition. Diverse nitrogen utilization pathways allow YL28 to grow on the different nitrogen compounds. YL28 can convert nitrite (toxic compounds in the mangrove ecosystems) into non-toxicity or low-toxicity products by fermentative nitrate reduction or assimilation nitrate reduction pathways. Denitrification may also contribute to this process^5^.

In this study, our bioinformatics studies further show that purple bacteria have 6 nitrogen cycle pathways (Table 3). The alternative nitrogenase genes are frequently observed in purple bacteria. The genes (encoding two nitrogenase subunits) are highly conserved in many phyla of bacteria and archaea, suggesting that nitrogen fixation genes evolve once and subsequently spread by vertical in heritance or by HGT^9^. Nitrate/nitrite reduction-related genes were found only in the accessory genomes. The observation agrees with the previous studies that show that nitrate/nitrite is not preferred nitrogen sources^29^. The partial denitrification pathway was widely found in PNSB^30,31^. To best of our knowledge, our results first demonstrated that the partial denitrification pathway existed in PSB, suggesting that some PSB possibly also played important roles in the nitrogen cycle (e.g., *Rhodopseudomonas* and *Rhodobacter*). The partial denitrification pathway contributes to remove excess redox and mitigates the toxicity from the certain nitrogen oxide intermediates^20^. The fermentative nitrate reduction is less known in purple bacteria and assimilation nitrate reduction is only limited to a member of PNSB (*Rhodobacter capsulatus* E1F1). However, our results showed the fermentation nitrate reduction (DNRA) was possible occurred in purple bacteria, and assimilation nitrate reduction was in PSB.

The use of reduced sulfur compounds as electron donors for anoxygenic photosynthesis has been found in all groups of purple bacteria (PSB and PNSB). Remarkably, PSB utilize higher concentration reduced sulfur compounds and accumulated element sulfur inside the cells than PNSB^20,32,33^. Our previous studies showed that YL28 was capable of utilizing diverse reduce sulfur compound and depositing sulfur granules inside the cells. It demonstrated a higher tolerance (3.6 mM) to sulfide than PNSB (0.5–2 mM)^1,2^. YL28 has the diverse sulfur metabolism-related genes such as *sqr*, *dsr*, *sox*, *apr* and *sat*. The oxidation of sulfide and the reversed dissimilatory sulfite reduction (key enzyme gene *sqr* and *dsr*) possibly contribute to detoxification of toxic sulfide. Thiosulfate may be converted into S^0^ or sulfate by truncated Sox system. Moreover, an alternative pathway (key genes *apr* and *sat*) also allows to detoxifing the toxic sulfite. However, sulfite may not be directly converted into sulfate via a two-electron transfer because purple bacteria lack of the sulfite:cytochrome c oxidoreductase^33^. Without *soxC/soxD*, sulfane sulfur atom cannot be subsequently oxidized^34^. However, YL28 has *soxD*. The study on the complete sulfur oxidation in YL28 by *soxD* needs to be investigated. Some genes involved in classical sulfite metabolism were not detected in most purple bacteria^34^ (except for some freshwater PSB species such as *A*. *vinosum* DSM180)^35^. However, YL28 has both sulfide: quinone reductase gene (*sqr*, YL28 could directly oxidize S^2−^ to S^0^) and APS reductase gene (*apr*). This suggests that YL28 may oxidize SO_3_^2−^ to SO_4_^2−^ and reduce the sulfite toxicity to cells. Sulfite reductase gene (*dsr*) was observed in all of the selected PSB (not PNSB). Gene *dsr* may be a useful taxonomic or systematic marker for PSB phylogeny.

Our studies revealed that there was a distinctive difference in halo-tolerance traits between freshwater and salt-dependent species by the 16S rDNA, core or whole genome sequence analysis. The salt-dependent or salt-requiring species showed different halo-tolerance. For example, YL28 possesses unique stress response genes (*betA*, *duf* 2062, *mrp*, glucan and heavy metal response genes etc.). These genes possibly contribute to the tolerance to in high salinity environment^36,37^.

The size of genome, the numbers and size of GIs in PSB were smaller than those in PNSB, implying that PSB had more flexible response to environment change. A gene transfer agent (GTA) is an unusual bacteriophage-like element which transfers a random host genomic DNA fragments (4–14 kb in size) between closely related bacteria^38–40^. Genes involved in the photosynthesis may be horizontally transferred between the same phyla by GTA^41^. Our results revealed that GTA gene clusters including ICEs (the self-transmissible mobile genetic elements, integrative and conjugative elements) were presented in all examined genomes. PNSB may acquire more genes by HGT to survive in the niches. The flagellum biosynthesis and methyl accepting chemotaxis genes (such as *cheA*, *cheB* and *cheR*) were found in the larger GIs, implying that purple bacteria possibly employ a complex set of chemosensory pathways to swim towards carbon and nitrogen sources, light and/or oxygen^42^.

## Conclusion

This study provided a novel insight into the mechanisms of diverse nitrogen cycle, habitat-specificity and toxic nitrite utilization in YL28 and purple bacteria (including PSB and PNSB). Purple bacteria possess 6 nitrogen cycle pathways. The denitrification, complete assimilation nitrate reduction and fermentative nitrate reduction were first demonstrated in PSB. The fermentative nitrate reduction was possibly widely occurred in purple bacteria. YL28 possesses good ability to utilize toxic nitrite which is possibly linked to the combination of three nitrogen cycle pathways (denitrification, fermentative nitrate reduction and complete assimilation nitrate reduction). Collectively, the genes involved in diverse nitrogen cycles (6 pathways), sulfur cycles (3 pathways), unique salt-alkali tolerance, stress response as well as other traits contribute to bacterial adaptation to the mangrove habitat.

## Materials and Methods

### DNA extraction, genome sequencing and annotation

YL28 was isolated from an intertidal sediment of the inshore mangrove in Fujian, China^2^. Genomic DNA of YL28 was extracted using an TIANamp Bacterial DNA kit following the protocols recommended by the manufacturer. Genome sequencing was performed by Shanghai Majorbio Bio-Pharm Technology Co. (China) using the Illumina HiSeq2000 sequencer system with a 500 bp pair-end library. The reads were assembled using SOAPdenovo v2.04. Putative protein-encoding genes were identified using Glimmer 3.02^43^. Annotation was performed by BLAST +2.2.24 searching against databases, including the National Center for Biotechnology Information (NCBI), Clusters of Orthologous Groups of Proteins (COG), the Kyoto Encyclopedia of Genes and Genomes (KEGG) and Gene ontology (GO). The genome sequence of *M*. *gracile* YL-28 was deposited in the GenBank database under the accession number LSYU00000000^44^.

### Acquisition and re-annotation of the selected genome sequences

The genome sequences of other 35 sequenced purple bacteria were obtained from NCBI database which assessed level was complete. To avoid the possible deviations due to different annotation methods, we used Rapid Annotation using Subsystem Technology (RAST) server for reannotation^45^ of the selected genomes. Glimmer algorithm was used for gene calling.

### Phylogeny

Three different methods were used for constructing the phylogeny trees. The 16S rDNA sequences of the selected microorganisms were first used to infer phylogenies using the Neighbor-Joining (NJ) method of MEGA 6.0.6^46^. The core genomes for these selected genomes were next clustered by USEARCH and the phylogeny trees of core and accessory genomes were constructed by Neighbor-Joining algorithm using the BPGA-1.3^47^. The 36 genome sequences were aligned by the progressive MAUVE to generate a phylogenetic guide tree^48^.

### Comparisons of conserved and variable regions

The genome of YL28 was used as a reference sequence to align all other genomes of 35 purple bacteria. Similarly, the genome of *R*. *palustris* CGA009 was selected as a reference to align those in *Rhodopseudomonas*, *Blastochloris* and *Rhodoplanes*; the genome of *R*. *sphaeroides* 2.4.1 was used as a reference to align the genomes of two genera *Rhodobacter* and *Rhodovulum*; *R*. *rubrum* ATCC11170 was selected as a reference to align the genomes of *Rhodospirillum* genus. The multiple whole–genome alignment was conducted using the progressive alignment algorithm implemented in MAUVE. Syntheny plots were generated by aligning regions of the predicted and reference genomes that differed by default parameter. All regions were aligned and displayed in MAUVE version 2.3.1^48^. The circular genomic map was generated by the BLAST Ring Image Generator (BRIG, version 0.95)^49^ using alignment reference genome on a local BLAST + basis, with standard parameters (50% lower – 70% upper cut-off for identity and E-value of 10). The ring color gradients correspond to varying degrees of identity of BLAST matches. Circular genomic maps also include the information on GC skew and GC content.

### Comparative analyses of core genome, accessory genome and unique genome

In order to depict the core and accessory genome in each genus, a reciprocal best hit search using the BPGA software was performed^47^. Orthologous clusters (OCs) were assigned by grouping all protein sequences in the 36 genomes using USEARCH based on their sequence similarity (E-value < 10^−5^, >50% coverage). A series of built-in scripts were used to (i) parse, (ii) upload to the MySQL relational database, (iii) perform a reciprocal best hit analysis to form pairs of sequences, and (iv) normalize the E-values for all the pairs formed. Normalization of E-values was done by averaging all recent ortholog (in paralogs) and dividing each pair of ortholog by the average. Pan-core plot against combinations will give core and pan genome boxplot and dot plot generated using desired number of unique combinations of genomes. Atypical GC analysis will give sequences of core, accessory and unique genes with atypical (extreme) GC content. COG and KEGG distributions of the core, accessory and unique gene families were calculated based on representative sequences. Keywords were used to query for nitrogen metabolism functional in the orthologous families and to calculate the number of matches of those functions, using custom bash commands.

### Genomic island prediction

Genomic islands (GIs) were predicted by using IslandViewer3^50^ including IslandPick, IslandPath-DIMOB and SIGI-HMM.

### Nitrogen metabolism, sulfur metabolism and stress response analysis

We chose three RAST subsystems for further analyze the nitrogen metabolism, sulfur metabolism and stress response pathways. The key protein sequences^51–53^ responsible for nitrogen metabolism, sulfur metabolism was used as reference sequences to search against the 36 genomes of purple bacteria by BlastP. The obtained sequence by BLAST was further evaluated by the combination of KEGG, RAST, CDART (conserved domain architecture retrieval tool^54^ and CDD (the conserved domain database)^55^.

## Electronic supplementary material

Supplementary figures

Supplementary Table 1

Supplementary Table 2

Supplementary Table 3

Supplementary Table 4
