# Supplementary material for: Comparative genome analysis of marine purple sulfur bacterium Marichromatium gracile YL28 reveals the diverse nitrogen cycle mechanisms and habitat-specific traits
Source: Sci Rep. 2018 Dec 13;8:17803. doi: 10.1038/s41598-018-36160-2 (PMC6292899; doi:10.1038/s41598-018-36160-2)
Supplement: Supplementary file 1 — Supplementary figures [file 41598_2018_36160_MOESM1_ESM.pdf]

Comparative genome analysis of marine purple sulfur bacterium *Marichromatium gracile* YL28 reveals the diverse nitrogen cycle mechanisms and habitat-specific traits

Bitong Zhu[1], Xiaobo Zhang [1], Chungui Zhao [1], Shicheng Chen [2], Suping Yang[1]

Supplementary Figures

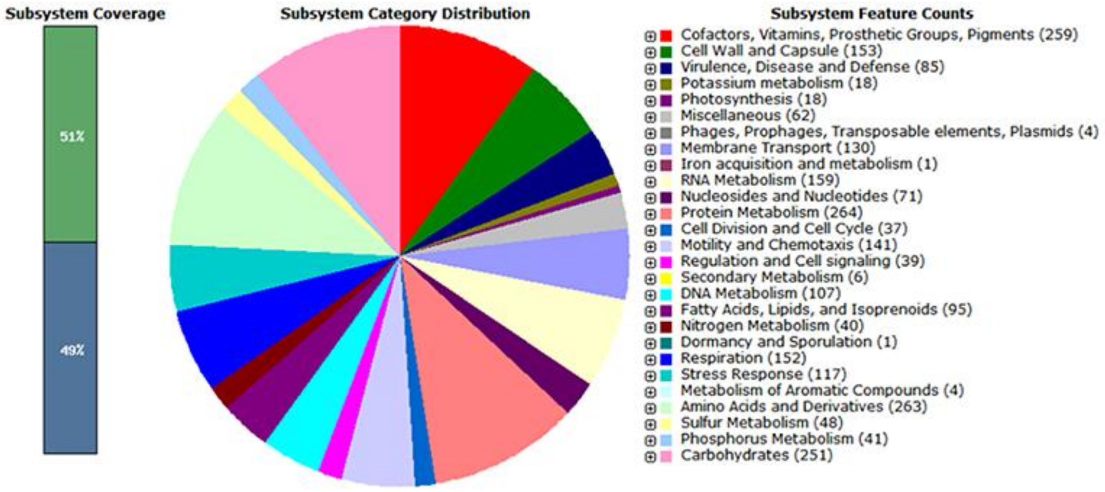

Supplementary Figure SF1. Subsystem statistics of *Marichromatium gracile* YL28. The subsystem category distribution shows the green column in the left (which is the 51% subsystem coverage), its color corresponds to the subsystem feature counts. The gray column in subsystem coverage is mainly

[1] Department of Bioengineering and Biotechnology, Huaqiao University, Xiamen 361021, China.
[2] Department of Microbiology and Molecular Genetics, Michigan State University, East Lansing, Michigan 48863, USA. Correspondence and requests for materials should be addressed to S.C. (shicheng@msu.edu) or S. Y. (yangsuping@hqu.edu.cn).

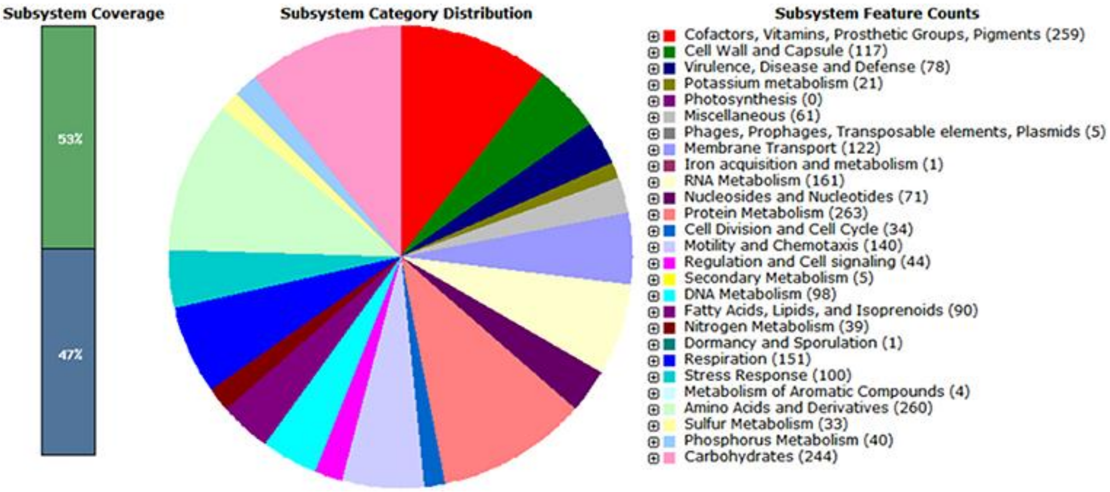

Supplementary Figure SF2. Subsystem statistics of *Marichromatium purpuratum* 984. The subsystem category distribution shows the green column in the left (which is the 53% subsystem coverage), its color corresponds to the subsystem feature counts. The gray column in subsystem coverage is mainly.

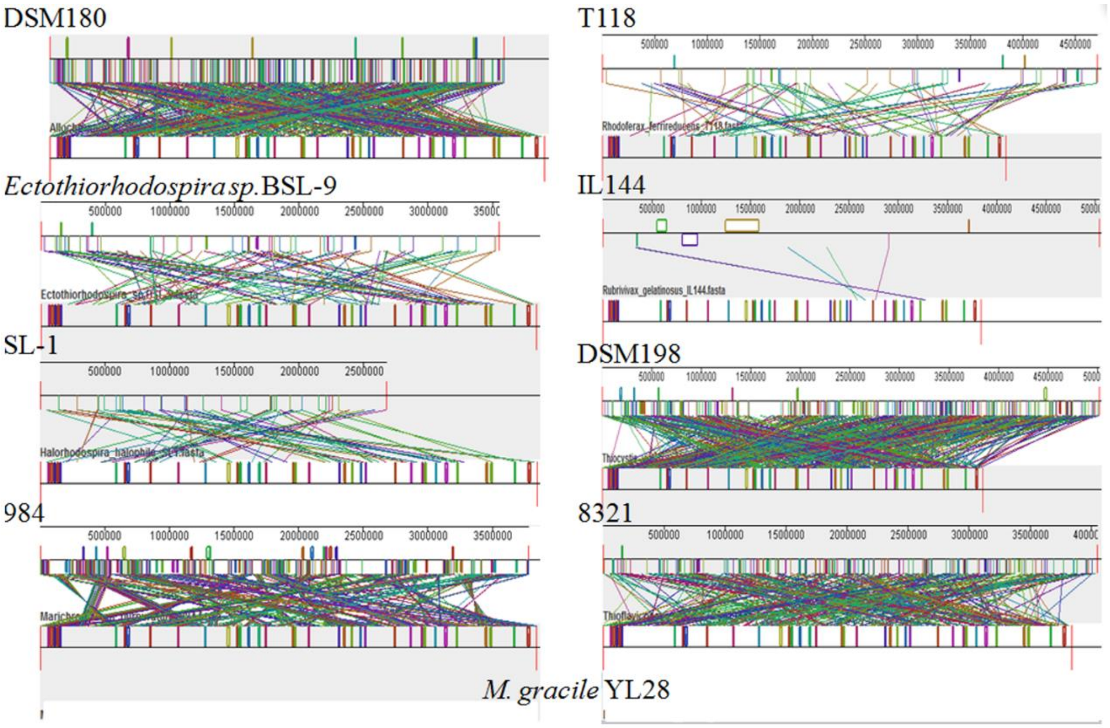

Supplementary figure SF3. Synteny plots of the genus *Rhodoferax, Rubrivivax*, PSB strains against reference strain *M. gracile* YL28.

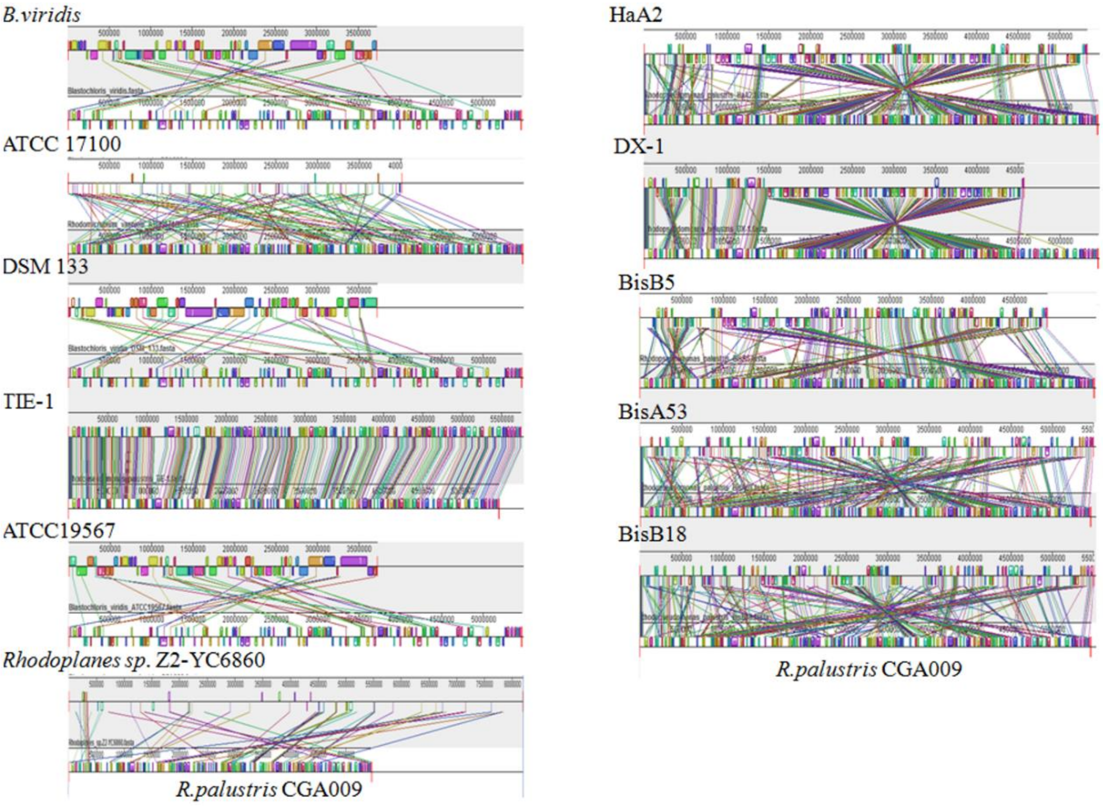

Supplementary figure SF4. Synteny plots of the genus *Blastochloris*, *Rhodopseudomonas*, *Rhodoplanes*, *Rhodomicrobium* strains against reference strain *R. palustris* CGA009

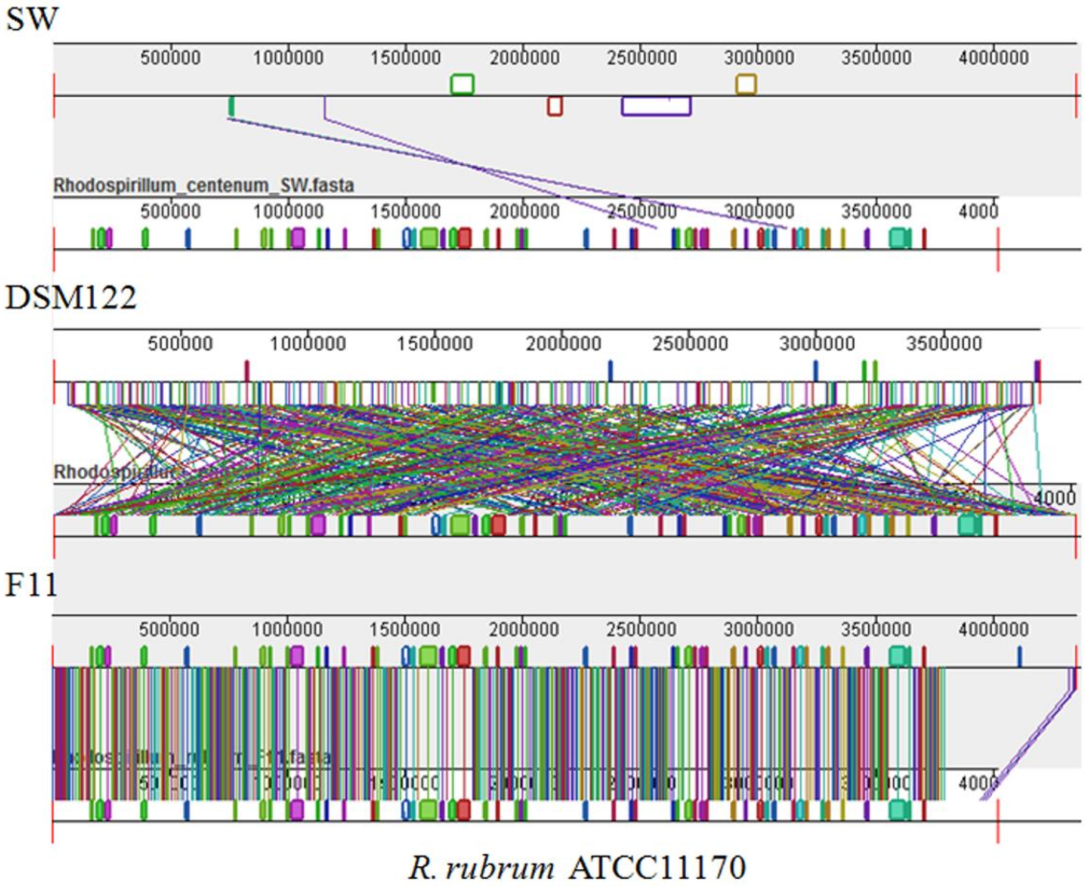

Supplementary figure SF5. Synteny plots of the genus *Rhodobacter*, *Rodovulum* strains against reference strain *R. sphaeroides* 2.4.1.

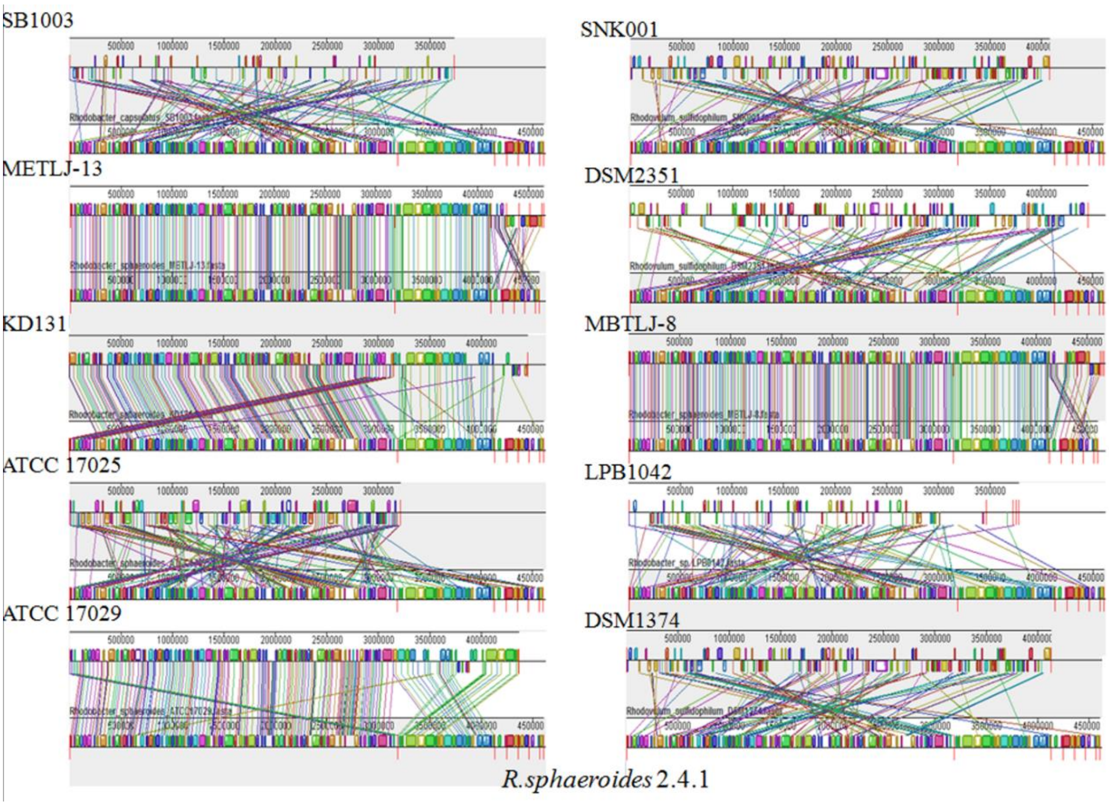

Supplementary figure SF6. Synteny plots of the genus *Rhodospirillum* strains against reference strain *R. rubrum* ATCC11170.
